# From Assessment to Implementation: Innovations to Decreasing Dementia Risk in Rural Settings

**DOI:** 10.1093/geroni/igaf122.1596

**Published:** 2025-12-31

**Authors:** Lisa Wiese, Heather Fuller, Cassandra Ford

## Abstract

Though rural U.S. counties comprise 85% of the “older-age counties,” where more than 20% of residents are >65, innovative interventions to minimize the age-related risk of dementia are not well established. This symposium offers advances in assessment, recruitment, data collection, and interventions among rural U.S. regions. Fergen and colleagues share findings from their approach of using merged ethnographic and socioecological frameworks to identify dementia-focused themes that can be applied when developing interventions across the home, clinic, and community in rural northern Minnesota. Second, Cook et al. reveals new findings regarding the intersection of social isolation and environment in the rural community, through novel smartwatch-based measures of socialization and physical activity and how they relate to cognitive performance in older racially/ethnically diverse adults living in a rural southern Florida farmworker region. Third, Ghazi and colleagues compare the level of dementia risk as measured by the Montreal Cognitive Assessment (MoCA) across rural, micropolitan, and urban samples as a means for informing future innovations. Wiese’s team concludes the symposium by demonstrating the effectiveness of adapting a faith-based framework for cancer detection and diagnosis, to a new focus area; cognitive decline. This model, tested in a southern, rural, racially/ethnically diverse cohort, can serve as an upstream approach to increase primary (education), secondary (screening), and tertiary (treatment) of dementia in other rural settings. Finally, as discussant, Ford will synthesize implications of these findings for policy and practice. Combined, these abstracts provide a pathway for diminishing the threat of cognitive decline in rural communities.

